# Facial feedback affects valence judgments of dynamic and static emotional expressions

**DOI:** 10.3389/fpsyg.2015.00291

**Published:** 2015-03-17

**Authors:** Sylwia Hyniewska, Wataru Sato

**Affiliations:** The Hakubi Project, Primate Research Institute, Kyoto UniversityInuyama, Japan

**Keywords:** facial feedback, dynamic expression, emotion recognition, facial expression, dimensional rating

## Abstract

The ability to judge others' emotions is required for the establishment and maintenance of smooth interactions in a community. Several lines of evidence suggest that the attribution of meaning to a face is influenced by the facial actions produced by an observer during the observation of a face. However, empirical studies testing causal relationships between observers' facial actions and emotion judgments have reported mixed findings. This issue was investigated by measuring emotion judgments in terms of valence and arousal dimensions while comparing dynamic vs. static presentations of facial expressions. We presented pictures and videos of facial expressions of anger and happiness. Participants (*N* = 36) were asked to differentiate between the gender of faces by activating the corrugator supercilii muscle (brow lowering) and zygomaticus major muscle (cheek raising). They were also asked to evaluate the internal states of the stimuli using the affect grid while maintaining the facial action until they finished responding. The cheek raising condition increased the attributed valence scores compared with the brow-lowering condition. This effect of facial actions was observed for static as well as for dynamic facial expressions. These data suggest that facial feedback mechanisms contribute to the judgment of the valence of emotional facial expressions.

## Introduction

Judging the emotions that others are experiencing is an important skill in managing interpersonal relationships. Given that emotions guide behaviors (Frijda, 2010), understanding others' emotions allows to predict their behaviors and to coordinate social relationships. In fact, evaluating the emotional content of any behavior is essential in all social encounters, starting with basic judgments of the extent to which an ongoing event is attractive or aversive to another individual (Russell, 1994; Widen, 2013).

Several lines of evidence suggest that emotion judgment is modulated through such behavior as the mimicry of observed facial expressions. It has long been known that humans have a tendency to spontaneously imitate the expressions of others (Smith, 1759/1976), and experimental psychological studies have provided empirical evidence that the simple viewing of facial emotional expressions leads to the reproduction of similar expressions by viewers (e.g., Dimberg, 1982). Several researchers have proposed that the facial actions resulting from such mimicry influence emotion judgment via the feedback effect (Hatfield et al., 1994; Goldman and Sripada, 2005; Niedenthal et al., 2010). Specifically, researchers have suggested that muscle activations in response to others' emotional facial expressions provide feedback to the brain in the form of proprioceptive signals, which activate the representation of one's own emotional bodily state; this representation leads to understanding the emotions experienced by other people (Hatfield et al., 1994; Goldman and Sripada, 2005; Niedenthal et al., 2010). Neuroscientific research supports such ideas by showing that the mutual influence of the production and observation of expressions can be explained by a shared neural substrate, the mirror neuron system (Grezes and Decety, 2001; Atkinson and Adolphs, 2005; Iacoboni, 2009). Thus, the influence of facial feedback on the interpretation of the emotional expressions of others can be explained theoretically.

However, empirical investigations of the causal relationship between the facial actions of observers and the judgment of emotions have reported mixed findings. Several studies reported results supporting this relationship using designs involving the manipulation of facial actions with instruments (Niedenthal et al., 2001; Oberman et al., 2007; Ponari et al., 2012; Rychlowska et al., 2014), cosmetic procedures (Neal and Chartrand, 2011), and instructions (Stel and van Knippenberg, 2008). For example, Niedenthal et al. (2001) showed that participants whose spontaneous facial actions were disrupted by holding a pen in their mouth were slower to detect a change from one expression to another compared with participants who were free to react with their facial muscles. Neal and Chartrand (2011) found that limiting facial mimicry by injecting Botox into faces and amplifying the subjective experience of facial actions by applying gel to faces impaired and improved, respectively, emotion recognition based on facial expressions. However, some of these studies only partially supported this relationship. For example, Oberman et al. (2007), who also used a pen-holding technique to constrain the facial actions of observers, reported that this disruption impaired the emotion-labeling performance in response to some but not all emotions. Stel and van Knippenberg (2008) showed that constraining facial actions by asking participants not to move their faces reduced the speed but not the accuracy with which the emotion depicted in facial expressions was recognized. Furthermore, several studies that tested the correlation between the degree of facial mimicry and the accuracy of expression recognition found no evidence of such a relationship (Blairy et al., 1999; Hess and Blairy, 2001; however, see Sato et al., 2013). Following those findings, a number of researchers (Blairy et al., 1999; Hess and Blairy, 2001; Hess and Fischer, 2013) pointed out that whether the facial actions of observers modulate judgments of perceived emotional expressions in unrestricted conditions remains unclear.

Two factors seem to be important in order to clarify this issue: the use of dimensional measures to evaluate emotion and the use of dynamic vs. static presentations of facial expressions.

First, although all previous studies tested facial emotion judgments using emotional categories (e.g., anger), facial emotion can be interpreted using dimensions of valence and arousal. These dimensions are superordinate to categories (Russell, 2003), and the most prevalent interpretation of them is that valence, which ranges from negative to positive, represents the qualitative component, whereas arousal, which ranges from low to high, reflects the energy level (Russell, 2003). It has been proposed that dimensional judgments of facial expressions may be more fundamental than categorical ones (Russell et al., 2003). Several studies have supported this notion; for example, preschoolers order facial expressions in a two-dimensional space of valence and arousal without the use of emotion labels, as these seem not to be readily available at this stage of development (Russell and Bullock, 1986). Based on these data, we could argue that the unconscious feedback from the face, which is not explicitly related to an ongoing evaluative task and acts on a basic and non-verbal level, would be more clearly related to the dimensional attribution stage of facial expression judgments. Consistent with this notion, a recent study found a significant correlation between facial mimicry and emotion recognition using dimensional, specifically valence, ratings (Sato et al., 2013). Based on these findings, we hypothesized that facial actions would have a clear effect on emotion judgments made with dimensional valence ratings.

Second, although none of the previous studies compared dynamic and static presentations of facial expressions, this difference may modulate the facial feedback effect on emotion recognition. Previous psychological studies have shown that, compared with static facial expressions, dynamic ones facilitate various types of psychological activities, including facial mimicry (Weyers et al., 2006; Sato et al., 2008; Rymarczyk et al., 2011), subjective emotional arousal (Sato and Yoshikawa, 2007a), and emotion recognition (Wehrle et al., 2000; Biele and Grabowska, 2006). Functional neuroimaging studies have also shown that dynamic vs. static facial expressions enhanced activity in the mirror neuron system (Sato et al., 2004). Based on these data, we hypothesized that facial action would influence ratings of static and dynamic presentations of facial expressions and exert a stronger impact in reaction to dynamic presentations.

To test these hypotheses, we investigated the effect of facial actions on emotional evaluations offered in terms of valence and arousal ratings of dynamic and static facial expressions. To manipulate participants' facial actions, we used the voluntary facial action technique (Dimberg and Söderkvist, 2010), which requires participants to lower their brows (corrugator supercilii muscle) or raise their cheeks (zygomaticus major muscle) to differentiate between two types of stimuli; in our study, it was the gender of the stimuli that differed. This technique has been shown to be effective in the modulation of the valence of the subjective emotion reported while viewing emotional facial expressions in situations in which participants are not aware that the purpose of the experiment involves examining the effect of facial action on emotional processing (Dimberg and Söderkvist, 2010). We also prepared a cover story and a dummy task, to be administered before the actual facial action task, to hide the experimental purpose. We presented facial expressions of anger and happiness because (1) the voluntary facial action technique can elicit mimicry-like facial actions in response to these expressions and (2) correlations between facial actions and valence evaluations have been reported for these expressions (Sato et al., 2013).

## Materials and methods

### Participants

Thirty-six students from Kyoto University (15 females, 21 males, mean ± SD age, 22.1 ± 2.1 years) participated in this study. All participants had normal or corrected-to-normal visual acuity. Although six additional volunteers participated in the study, their data were not analyzed due to their reported psychological problems or outlier ratings (>2 SD from the group mean). Participants signed a written informed consent form after the experimental procedures were explained. The study was approved by the local ethics committee of the Primate Research Institute, Kyoto University. Participants were reimbursed for their time and effort.

### Experimental design

We used a three-factorial within-participants design: observer's action (brow lowering, cheek raising) × stimulus emotion (anger, happiness) × stimulus presentation (dynamic, static). Valence and arousal scores were the two dependent variables.

### Stimuli

**Figure 1:**
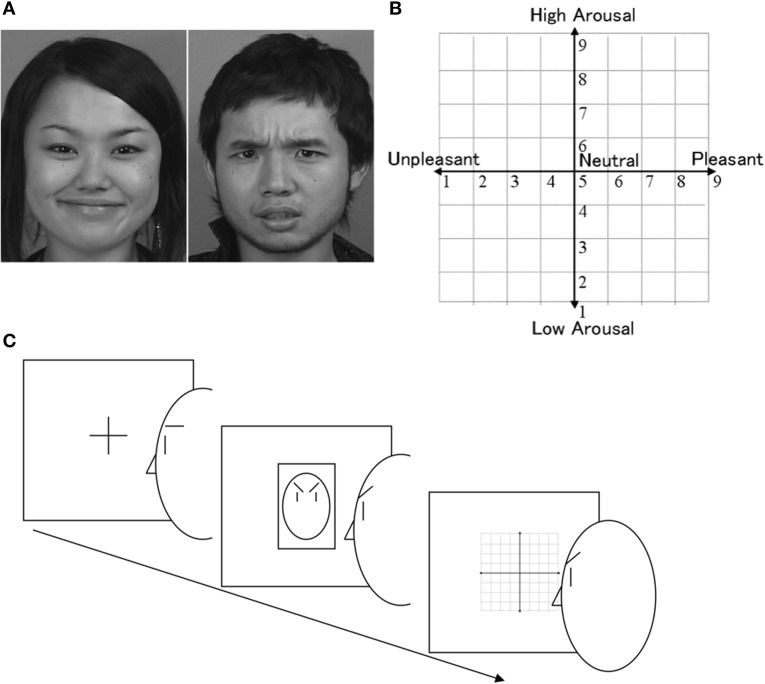
**Illustrations of materials and procedure**. **(A)** Examples of angry and happy expressions. **(B)** Affective grid provided to participants. **(C)** The sequences of a trial: (1) a fixation cross, (2) a facial expression stimulus and a participant's facial action, and (3) the evaluation of the stimulus.

The facial expressions (Figure 1A) were taken from the video corpus of emotional displays depicted by Kyoto University students (Sato and Yoshikawa, 2007b). The selection and validation of the angry and happy expressions in dynamic and static styles were described in a previous study (Sato and Yoshikawa, 2007b), which found high levels of accuracy in the recognition of these expressions by participants. Static pictures showed the peak expression in the video displays. Four displays of each emotion were chosen (the expressions of two male and two female actors). A dummy task, which preceded the one of interest, involved the presentation of pictures of robots and animals. Each stimulus subtended a visual angle of about 7.8° horizontally × 9.8° vertically. The viewing distance was approximately 0.7 m.

### Apparatus

The presentation of stimuli was controlled by Presentation® software version 14.9 (Neurobehavioral Systems) implemented on a Windows computer (HP Z200 SFF, Hewlett-Packard). The stimuli were presented on a 19-inches CRT monitor (HM903D-A, Iiyama). The facial actions of participants were monitored through a hidden digital camera (QuickCam IM, Logitech).

### Procedure

Participants were led individually to a sound-attenuated experimental room. As part of a cover story to reduce awareness of the focus of the research, a plethysmograph device was attached to the non-dominant hand of participants, and participants were told it would measure their heartbeat during the entire experiment. Participants then relaxed for 3 min.

Computer guidelines about the cover story and procedures were provided to participants. The first guideline indicated that the aim of the study was to investigate the practical use of technology by handicapped persons. Participants were told that they would be assigned to perform two tasks that were randomly chosen from a wide range of possible tasks; however, the same tasks were actually assigned to all participants. Participants were asked to evaluate the internal state of the stimuli by pressing keys to respond to an affect grid (Russell et al., 1989), which graphically represented the two dimensions of valence, from unpleasant (1) to pleasant (9), and of arousal, from low arousal (1) to high arousal (9) (Figure 1B). Following Russell et al. (1989), the midpoint of each scale was explained as representing a neutral, average feeling, whereas the vertices were defined as representing extreme emotions, such as excitement and depression.

In the dummy task, participants performed shoulder actions in response to the photographs of robots and animals. They were asked to move their left and right shoulder forward as fast as possible in response to robots and animals, respectively, and to evaluate the internal states of the stimuli using the affect grid. They were asked to hold the shoulder position until they finished responding. After a few practice trials for actions and for ratings with actions, a total of 12 trials, consisting of six trials each with robots and animals, were conducted. The order of trials was randomized. Each trial consisted of the presentation of a fixation cross for 500 ms; this was followed by the presentation of the stimulus for 1500 ms and then by the presentation of the affect grid. The inter-trial interval was 1000 ms. The results from the dummy task are not reported as the performance on this task was irrelevant to the purpose of the study.

In the experimental task (Figure 1C), participants performed facial actions in response to emotional facial expressions. They were asked to lower their brows and raise their cheeks as fast as possible in response to women and men, respectively, under one condition and to perform the facial actions in the opposite direction under another condition. They were also asked to evaluate the internal states of the stimuli using the affect grid while maintaining the facial action until they finished responding. The participants engaged in a few practice trials for actions and for ratings with actions. During the practice, participants were observed through a hidden camera by an experimenter certified in the use of the Facial Action Coding System (FACS: Ekman et al., 2002) to ensure the correctness of their facial actions according to this system. If the participant did not perform the facial actions appropriately (i.e., Action Units 4 and 12 for brow lowering and cheek raising, respectively), the experimenter corrected the actions by explaining that the plethysmograph device was not able to accurately detect the responses. The experimenter pointed either to brows or to cheeks, asking the participant if he/she could reproduce the expression presented on the screen while making it herself. No affective terminology was used to describe the facial action, nor were any related terms, such as “frown” or “smile,” used. One intervention was sufficient to correct facial actions during the experimental task. The participants completed a total of 64 trials presented in two blocks of 32. In one block, participants were asked to lower their brows when seeing women; in the other, they were asked to do so when seeing men (and the reverse for the cheek raising). The same stimuli were used in both blocks. The event sequence of each trial was the same as that in the dummy task (i.e., a fixation for 500 ms, the stimulus for 1500 ms, and then the affect grid).

After the experiment, the participants were interviewed. This process confirmed that no-one was aware of the purpose of our experiment. Participants were then debriefed regarding the experiment. Permission to use their data was requested and granted in all cases.

### Data analysis

Repeated-measures analyses of variance (ANOVAs) were performed treating observer's action (cheek or brow activation), stimulus emotion (happiness or anger), and stimulus presentation (dynamic or static) as factors. Valence and arousal were analyzed separately. Our effect of interest was the observer's action. When this factor showed significance, we further tested for simple effects under each stimulus condition using *t*-tests (one-tailed). The simple effects of other factors were also examined using *t*-tests (two-tailed). Based on our preliminary analyses, the gender of the participants, which showed no significant main or interactive effects on the results, was disregarded in the following analyses. The results of all tests were considered statistically significant at *p* < 0.05.

## Results

**Figure 2:**
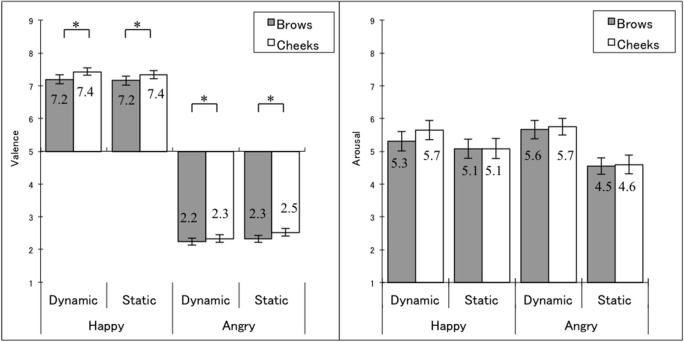
**Participants' valence and arousal scores**. Mean (with SE) valence scores **(left)** and arousal scores **(right)** attributed by participants to stimuli under eight experimental conditions. The valence scale ranged from unpleasant (1) to pleasant (9). The arousal scale ranged from low arousal (1) to high arousal (9). Asterisks indicate significant simple effects of the observer's actions (cheek raising > brow lowering).

In terms of valence scores (Figure 2 left; see Supplementary Figure 1 left for different scores between cheek raising and brow lowering conditions), the three-way ANOVA revealed a main effect of the observer's action, *F*_(1, 35)_ = 10.34, *MSE* = 0.24, *p* < 0.005, η^2^_*p*_ = 0.228, with more positive scores under the cheek raising compared with the brow-lowering condition. Simple-effect analyses confirmed that the effects of observers' action (cheek raising > brow lowering) were significant for all the dynamic happy, *t*_(35)_ = 2.12, *p* < 0.05, static happy, *t*_(35)_ = 1.95, *p* < 0.05, dynamic angry, *t*_(35)_ = 1.84, *p* < 0.05, and static angry expressions, *t*_(35)_ = 3.31, *p* < 0.005. We found no significant interactions related to the observers' action, *F*_(1, 35)_ < 1.18, *p* > 0.1. Additionally, the main effect of the stimulus emotion (happiness > anger), *F*_(1, 35)_ = 571.36, *MSE* = 3.11, *p* < 0.001, η^2^_*p*_ = 942, and the interaction between the stimulus emotion and the stimulus presentation, *F*_(1, 35)_ = 8.22 *MSE* = 0.10, *p* < 0.005, η^2^_*p*_ = 0.190, were significant. Simple effect analyses for the interaction revealed that the effect of stimulus emotion (happiness > anger) were significant both for dynamic and static presentations, *t*_(35)_ > 22.03, *p* < 0.001, and the effect of stimulus presentation (static > dynamic) was significant for angry, *t*_(35)_ = 2.93, *p* < 0.01, but not for happy expressions, *t*_(35)_ = 1.11, *p* > 0.1. The main effect of the stimulus presentation was not significant, *F*_(1, 35)_ = 2.72, *p* > 0.1.

In terms of arousal (Figure 2 right, Supplementary Figure 1 right), the three-way ANOVA showed no significant main effect or interactions related to the observers' action, *F*_(1, 35)_ < 2.27, *p* > 0.1. However, we found a significant main effect of stimulus presentation (dynamic > static), *F*_(1, 35)_ = 12.32, *MSE* = 3.58, *p* < 0.005, η^2^_*p*_ = 0.260, and a significant interaction between the stimulus emotion and the stimulus presentation, *F*_(1, 35)_ = 19.22, *MSE* = 0.57, *p* < 0.001, η^2^_*p*_ = 0.354. Simple effect analyses for the interaction revealed that the effect of stimulus emotion was not significant for either of dynamic or static presentations, *t*_(35)_ < 1.55, *p* > 0.1, and the effect of stimulus presentation (dynamic > static) was significant for angry, *t*_(35)_ = 4.23, *p* < 0.001, and marginally significant for happy expressions, *t*_(35)_ = 1.99, *p* < 0.1. The main effect of the stimulus emotion was not significant, *F*_(1, 35)_ = 0.62, *p* > 0.1.

## Discussion

Consistent with our first hypothesis, our results showed that observers' facial action had an impact on the valence ratings of stimulus facial expressions. Specifically, cheek raising led to higher valence scores for facial expressions than did brow lowering. These results are consistent with several previous studies that reported that the manipulation of facial actions by observers influenced emotion recognition (Niedenthal et al., 2001; Oberman et al., 2007; Neal and Chartrand, 2011). However, several studies reported cases in which facial action had no clear effect on the attribution of emotional labels to facial expressions (Oberman et al., 2007; Stel and van Knippenberg, 2008). Following these inconsistencies in categorical attributions to expressions, we relied on valence judgments, which have been defined as more fundamental than categorical judgments (Russell et al., 2003). Our experiment was the first to further test the facial feedback effect by using dimensional valence ratings, which seem even better able to detect consequent qualitative changes in judgments of the emotion of others.

With regard to our second hypothesis, the modulating effect of facial actions, cheek raising and brow lowering, was strong in response to static as well as to dynamic presentations. However, contrary to our expectations, the effect of facial action was equally strong in response to both presentation formats. This result is inconsistent with previous data showing that dynamic facial expressions were better able to elicit facial mimicry, subjective emotion, and emotion recognition than were static ones (e.g., Sato and Yoshikawa, 2007a). Consistent with most data regarding the effect of dynamic presentations (e.g., Detenber et al., 1998; Sato and Yoshikawa, 2007a), our data showed that dynamic stimuli were rated as more arousing than were static ones, therefore we expect that our dynamic stimuli would have elicited a stronger emotional impact than our static stimuli similarly to what was observed in the previous studies. One possible interpretation of the observed discrepancy concerns our request that participants voluntarily and clearly perform facial actions in response to both dynamic and static facial expressions; this manipulation may have induced the same feedback for both types of presentation. It is possible that the recognition of dynamic facial expressions is enhanced in natural settings due to the stronger facial mimicry than the one experienced in response to static facial expressions.

Our results showing a clear facial feedback effect on the valence attributed to facial expressions may have theoretical implications. The extant literature regarding facial mimicry has long assumed that the feedback effect of facial actions would play a fundamental role in expression recognition (Hatfield et al., 1994). Experimental evidence has supported the importance of facial mimicry in the processing of facial expressions, showing that facial mimicry occurs rapidly, even before conscious awareness of faces (Dimberg et al., 2000), and that it is elicited at developmentally early stages, even in newborn infants (Meltzoff and Moore, 1977). However, the specific information about others' emotional expressions provided by the facial feedback effect remained unknown. In the literature on the facial expression recognition, it was proposed that a dimensional evaluation is fundamental to this process (Russell et al., 2003). This notion has been supported by empirical evidence that the valence of facial expressions is processed rapidly, before conscious awareness of faces (Murphy and Zajonc, 1993), and that it is recognized at developmentally early stages, such as 2 years of age (Russell and Bullock, 1986). However, the mechanism underpinning the ways in which the valence of expressions can be recognized also remained unknown. Our results connect these bodies of literature and suggest that facial feedback plays a fundamental role in emotion recognition by providing information about the valence of facial expressions.

Our results may also have practical implications. Using an experimental approach, we showed the effectiveness of the voluntary facial action technique (Dimberg and Söderkvist, 2010) for eliciting the facial feedback effect on the judgments of emotional expressions. This easy and non-intrusive method may be used in ecological settings to assist in the judgments of others' emotions. For example, it may be possible to utilize this method in individuals touched by psychiatric disorders involving impairments in emotional communication, such as the autism spectrum disorder (ASD). Individuals with ASD are characterized primarily by impaired recognition of emotional facial expressions (Hobson, 1993). Consistent with the notion of a facial feedback effect, a recent study revealed that individuals with ASD were impaired compared with typically developing controls in their ability to engage in spontaneous facial mimicry in response to others' emotional expressions (Yoshimura et al., in press). At the same time, this study showed that the ASD group was able to voluntarily imitate facial expressions in a manner comparable to the control group. Based on these data, we speculate that it may be possible to assist individuals with ASD in their valence judgments of facial expressions by applying the voluntary facial action technique in a way that is congruent with others' facial expressions. It would be interesting to explore such possibilities in future research.

In addition to the effect of observers' facial action, our results showed that dynamic presentations of facial expressions intensified the ratings of arousal as well as part of valence. The intensifying effect of dynamic presentations on arousal ratings is in line with previous studies reporting that the ratings of intensity (Biele and Grabowska, 2006) and subjectively experienced arousal (Sato and Yoshikawa, 2007a) were higher for dynamic than for static facial expressions and that the ratings of experienced arousal were higher for dynamic than for static emotional scenes (Detenber et al., 1998; Simons et al., 1999, 2000). The modulatory effect of dynamic presentations on valence ratings were also reported in some studies using scenery stimuli (Detenber et al., 1998; Simons et al., 2000). Together with these data, our results suggest that dynamic presentations have an intensifying effect on the dimensional evaluations of emotional facial expressions, independently of the effect of observer facial action.

Several limitations of the present study should be acknowledged. First, because we contrasted two facial actions, we could not conclude whether these facial actions increased or decreased the valence evaluations. This issue can be investigated by introducing a baseline situation, such as a condition or group without any predefined facial constraints. Clarification of this issue should increase our understanding of the phenomenon. Second, because we relied on only two basic emotions (cf. Ekman, 1992), questions about whether other valenced emotional expressions would show a similar effect involving facial feedback remains unanswered. Further studies should overcome this weakness by introducing expressions with other basic emotions (e.g., fear) or even complex emotions (e.g., excitement; cf. Yik et al., 2011).

In summary, our data showed an effect of facial action on valence judgments. When individuals activated the zygomaticus major muscle they attributed more positive valence to dynamic and static facial expressions than when they activated the corrugator supercilii muscle. These results suggest that facial feedback mechanisms contribute to the evaluation of the valence of emotional facial expressions.

## Author contributions

SH and WS were responsible for the conception and design of the study, data acquisition and analysis, the interpretation of results, and the writing of the manuscript.

### Conflict of interest statement

The authors declare that the research was conducted in the absence of any commercial or financial relationships that could be construed as a potential conflict of interest.

## Supplementary material

Supplementary Figure 1 **Differences in scores between the observer action conditions**. Mean (with SE) differences between cheek raising and brow lowering conditions for valence (left) and arousal (right).
